# The role of perioperative tapentadol in inguinal hernia repair: Implications for acute analgesia and chronic postoperative pain – a narrative review

**DOI:** 10.1007/s10029-026-03599-6

**Published:** 2026-03-16

**Authors:** M. Jurdičová, J. Fricová, V. Masopust, R. Rokyta, M. Stříteský, M. Anders

**Affiliations:** 1 3rd Faculty of Medicine, Department of Plastic Surgery, Charles University, University Hospital Královské Vinohrady, Prague, Czech Republic; 2 1 st Faculty of Medicine, Department of Anesthesiology, Resuscitation and Intensive Medicine, Pain Management Center, Charles University, General University Hospital, U Nemocnice 2, Prague 2, Prague, 128 00 Czech Republic; 3 Department of Neurosurgery, Military Faculty Hospital, Charles University, Prague, Czech Republic; 4 3rd Faculty of Medicine, Prague, Charles University, Prague, Czech Republic; 5 1 st Faculty of Medicine, Department of Anesthesiology, Resuscitation and Intensive Medicine, Pain Management Center, Charles University, General University Hospital, Prague, Czech Republic; 6 1 st Faculty of Medicine, Department of Psychiatry, Prague, Czech Republic, Charles University, General University Hospital, Prague, Czech Republic

**Keywords:** Tapentadol, Inguinal hernia, Chronic postoperative inguinal pain, Preemptive analgesia, Neuropathic pain, Hernia repair

## Abstract

**Background:**

Chronic postoperative inguinal pain remains one of the most frequent complications after inguinal hernia repair. Tapentadol, with its dual mechanism of µ-opioid receptor agonism and noradrenaline reuptake inhibition, offers potential advantages in controlling both nociceptive and neuropathic components of pain.

**Methods:**

This narrative review summarizes experimental, preclinical, and clinical evidence on the use of tapentadol in perioperative analgesia, with focus on inguinal hernia surgery and prevention of chronic postoperative inguinal pain (CPIP). Relevant randomized trials, observational studies, and mechanistic data were evaluated.

**Results:**

Available evidence suggests that tapentadol provides effective postoperative analgesia with a favorable gastrointestinal and central nervous system tolerability profile compared with traditional µ‑opioid agonists. Preemptive administration may reduce acute postoperative pain, opioid rescue requirements, and the risk of transition to chronic pain in high‑risk settings.

**Conclusion:**

Current evidence supports tapentadol as an effective component of perioperative analgesia in inguinal hernia surgery, particularly for reducing acute postoperative pain and opioid rescue requirements. However, its potential role in preventing chronic postoperative inguinal pain remains insufficiently established and should be considered hypothetical, pending confirmation from high-quality randomized controlled trials.

## Introduction

Inguinal hernia repair is a common surgical procedure with a modest postoperative level of pain. With a laparoscopic approach, the level of pain is even lower postoperatively [1]. Despite modern surgical techniques and pharmacological innovations, there is still a risk of developing chronic postoperative inguinal pain (CPIP), with an incidence ranging between 10% and 12% worldwide [2]. Effective postoperative pain management remains a significant clinical challenge, essential not only for rapid patient recovery and improved long-term outcomes but also for preventing chronic postoperative pain [3].

According to these findings, it is very important to manage postoperative pain successfully. Tapentadol is a relatively new opioid painkiller with a dual mechanism of action and therefore appears to be a promising tool for the treatment of acute, chronic, nociceptive, or neuropathic pain [4]. Its unique mechanism, combining µ-opioid receptor agonism and norepinephrine reuptake inhibition, makes it particularly effective for mixed pain conditions, which are common in postoperative settings [5].

The aim of this narrative review is to summarize available perioperative evidence on tapentadol use in hernia surgery, with a primary focus on acute postoperative analgesia and a secondary discussion of its potential implications for chronic postoperative inguinal pain.

## Methods

In this review, we analyzed 19 articles retrieved from Google Scholar, published between the years 2020 and 2025. The inclusion criteria required articles to be written in English, involve patients older than 18 years, and contain both the terms “inguinal hernia repair” and “tapentadol.” Additionally, tapentadol had to be included in the analgesic protocol either before or after hernia repair.

Articles that only mentioned tapentadol as a general recommendation for analgesic therapy, or lacked specific information about the timing of its administration, were excluded from the review. Only six studies met the inclusion criteria. None of the articles published between 1990 and 2025 in the Google Scholar and PubMed databases addressed tapentadol as a treatment for chronic groin pain following hernioplasty. However, one prospective study is currently investigating the use of tapentadol prolonged release (PALEXIA Retard 50 mg) to prevent chronic postoperative pain after inguinal hernia surgery and knee replacement surgery. The trial monitors patients for up to one-year post-surgery to evaluate whether tapentadol can effectively reduce the development of chronic pain [6]. This ongoing study highlights the growing interest in tapentadol’s potential for CPIP prevention, with preliminary results expected to contribute significantly to the field [6].

## Results

Vadhanan, in their study, administered 100 mg extended-release tablets of tapentadol orally to patients preoperatively. The control group received placebo tablets. Inguinal hernia surgeries were then performed under spinal anesthesia. The results showed reduced consumption of analgesics, a longer duration before the use of postoperative analgesics, and lower pain scores within 12 h after the procedure compared to the control group [7]. These findings suggest that preemptive tapentadol administration may reduce acute postoperative pain and the need for additional analgesics [7].

Similarly, Mitra published a randomized double-blind controlled trial comparing postoperative analgesic strategies using tapentadol–paracetamol versus tramadol–paracetamol in patients undergoing open hernioplasty. After the procedure, patients received either 100 mg of tramadol or tapentadol, along with 500 mg of paracetamol, administered orally. The trial found no significant differences between the groups. Overall, pain levels were lower after 4 h, the visual analogue scale indicated modest pain levels, and no clinical characteristics were statistically significant. There were also no statistically significant differences in adverse effects. The evidence suggests that both opioid analgesics, when combined with paracetamol, are similarly efficacious [8]. However, tapentadol’s dual mechanism may offer advantages in patients with neuropathic pain components, which are common in CPIP [9].

Among these studies, Alzatari compared non-opioid and opioid analgesics in patients undergoing ventral hernia repair. Patients’ pain scores, satisfaction with the procedure postoperatively, and outcomes one year after surgery were similar in both groups. However, on postoperative day 30, patients in the non-opioid group reported lower pain levels [10]. This suggests that while tapentadol is effective in the early postoperative period, non-opioid strategies may provide comparable long-term outcomes in some cases [10].

The remaining studies reached a consensus, supporting the general recommendation to use tapentadol as a second-line treatment for neuropathic and acute postoperative pain—not only following inguinal hernioplasty [11]. These studies emphasize tapentadol’s efficacy in managing mixed pain types, making it a valuable option for CPIP prevention [12].

The main findings of the trials are detailed in Table 1.

**Table 1:** Note: This data is mandatory. Please provide

Study (year)	Design	Population/Procedure	Intervention (timing)/Comparator	Primary endpoints	Key results (effect direction)	Notes
Vadhanan & Rajendran [7]	Randomized controlled trial	Inguinal hernia surgery under spinal anaesthesia	Tapentadol ER 100 mg preemptive vs. placebo	Early postoperative pain, time to first analgesic, rescue use	↓ pain at 6–12 h; ↑ time to first analgesic; ↓ rescue use	Supports preemptive tapentadol for acute analgesia
Mitra et al. [8]	Randomized comparative clinical study	Open hernia repair	Tapentadol 100 mg + paracetamol 500 mg post‑op vs. tramadol 100 mg + paracetamol 500 mg	Pain scores, adverse effects	Analgesia similar; lower nausea and better 12 h pain with tapentadol	Suggests tolerability advantage
Alzatari et al. [10]	Cohort (ventral hernia)	Ventral hernia repair	Opioid‑based vs. non‑opioid pathway	Pain (24 h, day 30), QoL (1 mo)	non‑opioid pathway: ↓ 24 h pain; ↑ QoL at 1 month	Favors opioid sparing protocols
Kelly et al. [13]	Retrospective	Abdominal surgery (mixed)	Institutional prescribing practices	Opioid exposure, antiemetic/analgesic variation	Wide variability (opioid 45–78%)	Highlights stewardship need
Kim et al. [14]	Narrative review	Chronic postsurgical pain	Multimodal prevention/management	Recommendations	Endorses multimodal, early intervention	Context for CPIP pathways
Szok et al. [11]	Review	Neuropathic pain (peripheral/central)	Pharmacologic/non pharmacologic options	Efficacy summaries	Mixed evidence; mechanistic rationale for MOR/NRI	Supports role in neuropathic components

*ER* extended release, *QoL* quality of life, *CPIP* chronic postoperative inguinal pain

Arrows: ↓ decrease; ↑ increase

## Discussion

According to the European Hernia Society (EHS) guidelines, chronic postoperative inguinal pain (CPIP) is defined as pain persisting for at least three months after surgery, which was not present before the operation or has different characteristics, and which impacts daily activities or quality of life [2]. CPIP is “pain above level 3 on the visual analogue scale, lasting at least 3 months postoperatively, that interferes with daily activities, is perceived as bothersome, and sometimes has neuropathic components” [15]. Within a year, the pain usually disappears, but up to 30% of patients continue to suffer from it permanently [15]. According EHS risk factors for developing CPIP include young age, female gender, high preoperative pain, early high postoperative pain, recurrent hernia, and open repair [2]. Clarke et al., in his study, also highlights the importance of an experienced and skilled surgeon in preventing chronic pain after surgical procedures [16]. Chu’s study further identifies the presence of other postoperative complications [OR = 1.849 (95% CI 1.034–3.305)], hernial sac defect < 3 cm [OR = 1.370 (95% CI 1.012–1.853)], and a history of ipsilateral inguinal hernia repair [OR = 2.706 (95% CI 1.445–5.069)] as significant risk factors for developing CPIP [17]. These risk factors underscore the need for tailored pain management strategies to mitigate CPIP development [17].

Management and treatment of CPIP are extremely challenging. First, the management of postoperative pain with adequate analgesics is crucial for preventing the development of chronic pain. It is recommended to combine general or spinal anesthesia with local wound infiltration using anesthetics such as trimecaine or bupivacaine. The combination of general and local anesthesia yields better outcomes, with significantly lower postoperative analgesic requirements compared to groups without local anesthesia. Local anesthesia may be administered via subcutaneous, subfascial, or periportal infiltration, or through a transversus abdominis plane (TAP) block. The TAP block is a promising technique that results in lower postoperative pain levels, delayed use of analgesics, reduced opioid dosages, and shorter duration of opioid use [18]. The integration of TAP block with tapentadol could further enhance pain control and reduce CPIP incidence [18].

Second, and this step is crucial, is proper postoperative pain management. A key strategy is to reduce pain using effective analgesics, such as a combination of paracetamol and metamizole with NSAIDs (e.g., ibuprofen, diclofenac, nimesulide) for moderate pain. In cases of persistent or severe pain, escalation to opioid analgesics is recommended [2]. Tapentadol’s role as a second-line opioid is particularly relevant here due to its favorable side effect profile and efficacy in neuropathic pain [19].

Chronic postoperative inguinal pain should initially be treated using non-surgical methods. This includes non-steroidal and opioid analgesics, comprising tramadol and stronger agents such as piritramide, oxycodone, morphine, and tapentadol, which appears to be a particularly promising option [2]. If non-surgical approaches fail, selective or triple-selective neurectomy or mesh removal may be necessary [20]. Chronic postoperative inguinal pain has been shown to negatively affect long-term outcomes after hernia repair and may be associated with an increased risk of reoperation, even when modern mesh techniques are used [21]. Tapentadol may represent a useful component of multimodal pain management strategies in patients with CPIP due to its efficacy in mixed nociceptive and neuropathic pain, although its impact on long-term outcomes remains uncertain [22].

Tapentadol functions as an opioid analgesic with a dual mechanism: it acts as a µ-receptor agonist and a norepinephrine reuptake inhibitor. In vivo studies in rats have shown that tapentadol inhibits glutamatergic synaptic transmission without affecting the postsynaptic membrane. This leads to reduced excitability in CA3 hippocampal pyramidal neurons, which are believed to play a role in nociceptive processing [23]. Michot’s study confirmed that tapentadol acts at both cephalic and extra-cephalic levels and suggested that another mechanism involves the downregulation of neuroinflammatory responses [24]. These mechanisms make tapentadol uniquely suited for addressing the neuropathic components of CPIP [9].

Recent literature supports tapentadol’s effectiveness in treating acute, nociceptive, and neuropathic pain [4]. Compared to other opioids, 50 mg of tapentadol is equivalent to 10 mg of oxycodone in pain relief. Tapentadol also offers advantages over other opioids. Due to the widespread distribution of µ-opioid receptors throughout the body, opioid use may cause side effects such as dysphoria, euphoria, sedation, constipation, endocrine suppression, cardiovascular issues, convulsions, nausea, vomiting, opioid-induced hyperalgesia, opioid-induced ventilatory impairment (OIVI), persistent postoperative opioid use (PPOU), and opioid misuse or diversion. Opioid consumption tends to be higher in patients with depression, anxiety, or fear of pain [19]. Tapentadol’s lower affinity for µ-opioid receptors and its norepinephrine reuptake inhibition contribute to a reduced risk of these adverse effects, making it a safer choice for long-term pain management [25].

Notably, the risk of developing OIVI and PPOU is significantly lower with tapentadol. Nausea and constipation also occur less frequently during treatment [19]. Tapentadol has demonstrated efficacy in relieving trigeminal neuropathic pain, diabetic peripheral neuropathy, chronic low back pain, and postoperative pain following orthopedic surgeries, particularly knee arthroplasty [26]. In cases of trigeminal neuropathy, withdrawal after 14 days of tapentadol treatment occurred without any reported side effects [26]. These findings support tapentadol’s role as a versatile analgesic for both acute and chronic pain scenarios, including CPIP [12].

While preemptive analgesia has been shown to reduce acute postoperative pain and analgesic consumption, direct evidence supporting a preventive effect of tapentadol on the development of chronic postoperative inguinal pain is currently lacking. Available studies primarily demonstrate benefits in early postoperative outcomes, whereas long-term effects on CPIP remain unclear. Therefore, any potential role of tapentadol in CPIP prevention should be interpreted with caution and regarded as hypothesis-generating rather than conclusive. Preemptive analgesia deserves particular attention. In the context of preemptive use, Vadhanan’s study describes a strong analgesic effect of tapentadol administered before hernia repair under spinal anesthesia [7]. Similar conclusions were reported by Fricová et al. (2009; 2010), who emphasized that preemptive administration of analgesics significantly reduces postoperative pain intensity, decreases the need for rescue opioid therapy, and improves functional recovery [15, 27, 28]. In line with Kim et al., preemptive analgesia should be framed as part of a comprehensive, multidisciplinary strategy for perioperative pain, while its long-term effects on CPIP remain to be clarified [14].

## Conclusion

The reviewed studies highlight the critical need for effective postoperative pain management to prevent chronic postoperative inguinal pain (CPIP) following inguinal hernia repair. Multimodal analgesia, incorporating preemptive strategies and a combination of non-opioid and opioid analgesics like tapentadol, has shown promising results in improving patient outcomes while minimizing adverse effects. Tapentadol’s dual mechanism of action, combining µ-opioid receptor agonism with norepinephrine reuptake inhibition, positions it as a particularly effective option for managing the complex pain profiles associated with CPIP, which often include both nociceptive and neuropathic components [4]. Its ability to reduce acute postoperative pain, as demonstrated in studies like Vadhanan et al. [7], and its favorable side effect profile compared to traditional opioids, such as lower risks of opioid-induced ventilatory impairment (OIVI) and persistent postoperative opioid use (PPOU), make it a valuable tool in the perioperative setting [19].

Furthermore, tapentadol’s efficacy in neuropathic pain conditions, such as trigeminal neuralgia and diabetic peripheral neuropathy, suggests its potential to address the neuropathic elements of CPIP, which are particularly challenging to manage [26]. The ongoing prospective study by Academisch Ziekenhuis Leiden (2025) investigating tapentadol’s role in preventing CPIP is a critical step toward establishing evidence-based protocols for its use in hernia surgery [6]. However, the variability in prescribing practices and the reliance on opioid-centric regimens underscore the urgent need for standardized guidelines to optimize pain management strategies. Future research should focus on large-scale, randomized controlled trials to further elucidate tapentadol’s long-term efficacy in preventing CPIP, particularly in high-risk patient groups identified by factors such as young age, female gender, and recurrent hernia [2]. Additionally, integrating tapentadol with other multimodal approaches, such as transversus abdominis plane (TAP) blocks, could enhance its effectiveness and further reduce the incidence of CPIP [18]. Ultimately, the adoption of individualized, evidence-based pain management protocols incorporating tapentadol could significantly improve patient recovery, reduce the burden of chronic pain, and enhance quality of life following inguinal hernia repair.
